# Evaluation of COVID-19 rapid antigen test against polymerase chain reaction test in immunocompromised patients

**DOI:** 10.1371/journal.pone.0306396

**Published:** 2024-08-02

**Authors:** Ali Sabateen, Dana Sadaqa, Taleen Nino, Ghayd Zaghal, George Qumsieh, Reena Fakhori, Hammam Rjoub, Tahreer Taha, Rami Zghari, Sari Abu Hanieh, Duaa Al-Basha, Marwan Qabaja, Hamza Alsaid, Musa Y. Hindiyeh

**Affiliations:** 1 Infectious Diseases Unit, Augusta Victoria Hospital, East Jerusalem, Palestine; 2 Molecular Laboratory, Augusta Victoria Hospital, East Jerusalem, Palestine; 3 Pharmacy Department, Modern University College (MUC), Ramallah, Palestine; 4 Pathology Laboratory, Augusta Victoria Hospital, East Jerusalem, Palestine; 5 Internal Medicine Department, Hadassah University Hospital, Jerusalem, Israel; Imam Abdulrahman Bin Faisal University, SAUDI ARABIA

## Abstract

On the 11^th^ of March 2020, the world faced a new global pandemic, COVID-19 which is a disease caused by the novel coronavirus, it had multiple devastating outcomes on multiple sectors along with significant rates of mortality. These challenges encouraged the development of multiple testing methods, as well as anti-viral medications such as Molnupiravir, as well as evaluating the efficacy of available medications against it, like; Azithromycin, Ritonavir and Hydroxychloroquine. Vaccination against COVID-19 forged into a significant challenge, few months ensuing the first case of SARS-CoV-2, which was diagnosed in December 2019, in Wuhan-China, thus, multiple vaccines were approved for use around the world to combat this pandemic. Our study includes a sample of 556 oncology patients at Augusta Victoria Hospital in Jerusalem, all patients were tested using Panbio rapid antigen test and Allplex PCR Assay. The main objective was to study the sensitivity and specificity of Rapid antigen test, which contributes to a faster isolation call and management of infected patients, thus decreasing the risk on spread to other patients and health care. Patients were categorized based on two factors: Ct range and age group and studying their possible effect on false-negative results. Patients with Ct value less than 20, had the highest detection rate which is consistent with other studies in the literature. The sensitivity and specificity of Panbio Rapid Antigen testing were of 69.9% and 100%, respectively. A correlation between age group and false negative results could not be made, but a correlation between Ct value and false negative result was noticed, Ct value was directly related to false negative results. P-value of 0.007 indicated that results were statistically significant where PCR test is considered more sensitive compared to rapid antigen test.

## Introduction

Starting in late December 2019, Wuhan, Hubei Province, the world faced the rapid and unprecedented spread of the devastating COVID-19 pandemic caused by severe-acute-respiratory-syndrome-related coronavirus (SARSr-CoV-2). Patients infected with the virus had a wide variety of symptoms, ranging from mild viral illness to severe ARDS or even death. Symptoms usually appeared 2–14 days after exposure to the virus [1]. SARS-CoV-2 became a threat to humanity thus rapid and sensitive diagnostic assays were needed to promptly diagnose infected patients and even health care providers, who were under significant exposure to the disease especially with rising incidence. Thus, rapid diagnostic assays with a short turnaround time from sample collection to results were needed to detect infected patients in healthcare setting to reduce hospital-acquired SARS-CoV-2 infections.

With the availability of viral genome sequencing, many molecular diagnostic assays were developed to detect the virus genome. Real-Time Quantitative Reverse Transcription Polymerase Chain Reaction (qRT-PCR) became the “Gold Standard” assay for the detection of SARS-CoV-2 Genome. Assays were developed to target multiple areas of SARS-CoV-2 genome to increase the sensitivity and specificity of the assays. In general, SARS-CoV-2 qRT-PCR assays have an average Turnaround Time (TAT) from sample collection to result reporting of 3–4 hours. Therefore, dedicated laboratory space, specialized equipment, and Medical Technologist expertise were needed to run the assays.

While SARS-CoV-2 qRT-PCR test was proved to be very crucial in controlling the virus outbreak, the need for diagnostic assays with faster turnaround time was clearly needed in particular to rapidly detect patients in hospital settings.

Many SARS-CoV-2 diagnostic assays with a short turnaround time of less than 30 minutes were developed and validated for diagnosing COVID-19. Of these, the SARS-CoV-2 antigen-based assays were developed to rapidly detect SARS-CoV-2 antigens in patient samples in less than 15 minutes. Abbott Panbio^TM^ COVID-19 Rapid Test Device (Panbio) is one assay that was developed to detect SARS-CoV-2 antigens in less than 15 minutes.

In this prospective study, we compared the performance of the Abbott Panbio^TM^, COVID-19 Rapid Test Device (Panbio) against Seegene Allplex™ SARS-CoV-2 qRT-PCR Assay (Allplex).

## Materials and methods

### Study design

This prospective study was conducted on 556 patients evaluated at Augusta Victoria Hospital (AVH) between January 2020 and June 2021. Patients’ age range was from 1 month to 90 years of age with an average age of 41.8 years. Of the 556 patients, 481 (86.5%) were adult patients and 75 (13.5%) were pediatric patients. with an overall male to female ratio of 1:1.04.

Patients arriving at AVH with any signs of respiratory symptoms, were simultaneously evaluated for the presence of SARS-CoV-2 antigens by Panbio^TM^ COVID-19 Ag Rapid Test Device and for the presence of SARS-CoV-2 genome by Seegene Allplex™ SARS-CoV-2 qRT-PCR Assay (COVID-19 RNA). Two nasopharyngeal samples (NPS), one from each nostril were collected; by well-trained nurses after donning the appropriate Personal Protective Equipment (PPE), according to the assays manufacturer recommendations. One NPS swab was collected using the Panbio^TM^ COVID-19 Ag Rapid Test Device provided swab and placed into the appropriate container per the recommendation of the manufacturer while the second swab was collected using Copan flocked swabs was placed in Copan UTM (universal transport medium) pending analysis.

#### Abbott Panbio^TM^ COVID-19 Rapid Test Device

This lateral flow assay (LFA) is intended to rapidly detect SARS-CoV-2 Antigen in the patient’s nasopharyngeal swab within 15 minutes. The Panbio^TM^ COVID-19 Ag Rapid Test Device is a chromatography assay that contains a membrane strip that is pre-coated with immobilized anti-SARS-CoV-2 antibody on the test line and mouse monoclonal anti-chicken IgY on the control line. Human IgG gold conjugate specific to SARS-CoV-2 Ag and chicken IgY gold conjugate move upwards on the membrane chromatographically and react with anti-SARS-CoV antibody and pre-coated mouse monoclonal anti-chicken IgY, respectively. The presence of SARS-CoV-2 antigen in the patient sample will be indicated by the development of a test line in the result window. A visible control line is required to indicate a valid reportable test result.

#### Total nucleic acid extraction

Total patient nucleic acid was extracted from 400μl of well-vortexed NPS sample using the Maglead^®^ 12gC (PSS, Japan). Nucleic acid was eluted in 100μl and stored at -70°C, pending analysis.

#### Seegene Allplex™ SARS-CoV-2 Assay

The FDA-EUO-approved multiplex qRT-PCR was designed to detect three SARS-CoV-2 target genes and one internal control gene in a single reaction tube within 3–4 hours. The assay detects SARS-CoV-2 specific RdRP and N genes and the E gene of all the *Sarbecovirus*, which also include SARS-CoV-2.

### Ethical approval

Approval of this research was waived by Augusta Victoria Hospital Ethical Committee, ethical IRB number; 25/GLD/2020. The patient’s written consent form was waived as the patients were informed that the two laboratory analyses would be performed anyway.

### Data analysis

A Microsoft Excel database was built, and used to analyze the data. Chi-square was used to determine the statistical significance of the data.

## Results

### Panbio^TM^ COVID-19 Test Device clinical sensitivity and specificity

Of the 556 patient’s analyzed NPS, 112 (20.1%) samples were positive by the Allplex^TM^ SARS-CoV-2 Assay, while 78 (16.3%) were positive by the Panbio^TM^ COVID-19 assay. Thirty-four samples were negative by Panbio^TM^ COVID-19 Ag Rapid Test Device and positive by the Allplex^TM^ SARS-CoV-2 Assay. Thus, the overall sensitivity, specificity, positive predictive value (PPV), and negative predictive value (NPV) were, 69.6%, 100%, 100%, and 92.9%, respectively.

### Panbio^TM^ COVID-19 Test Device detection limit based on qRT-PCR assay Ct value

Stratifying the positive SARS-CoV-2 samples by the qRT-PCR Ct value, revealed that the Panbio^TM^ COVID-19 Ag Rapid Test Device performed well with a high sensitivity (91.8%) when the qRT-PCR Ct value was below 20. The Panbio^TM^ COVID-19 Ag Rapid Test Device sensitivity dropped to 77.5% in patient samples with a Ct value 20–30. The Panbio^TM^ COVID-19 Ag Rapid Test Device performance was very poor in samples with qRT-PCR Ct values between 30–34 and 35–40, 18.2% and 0%, respectively.

### Distribution of false negative Panbio^TM^ COVID-19 Test Device results by age

To better understand if age played a role in the false negative results of the Panbio^TM^ COVID-19 Test Device, the Panbio^TM^ COVID-19 negative samples (N = 34) were stratified by age group as indicated in Table 1. Of the 34 false negative samples, 26.5% belonged to the 41–50 years of age, followed by 17.6% in the two age groups 31–40 and 41–60, respectively. The age groups 70–80 and 81–90 had 14.7% false negative Panbio^TM^ COVID-19 results. No difference was noted in the distribution of the qRT-PCR Ct ranges between the different age groups. Relationship between results was analyzed through Chi-square test and P-value was 0.007.

**Table 1:** Comparison between PCR and rapid antigen tests results, sensitivity, specificity, PPV and NPV of rapid antigen test.

		Rapid Antigen	
**PCR**		**Positive**	**Negative**
	**Positive**	78	34
	**Negative**	0	444
	**Total**	78	478
	**Sensitivity**	69.6	
	**Specificity**	100	
	**PPV**	100	
	**NPV**	92.9	

### Role of the utilization of the Panbio^TM^ COVID-19 Test Device in infection control

The rapid turnaround time of the Panbio^TM^ COVID-19 Ag Rapid Test Device allowed the infection control (IC) team to make quick decisions and move the infected patients to the COVID-19 center on 69.6% of the SARS-CoV-2 patients within 30 minutes of the patient’s arrival to the hospital avoiding causing SARS-CoV-2 hospital-acquired infections. However, the presence of negative Panbio^TM^ COVID-19 Ag Rapid Test in patients with very high viral titers (low Ct value) mandates keeping careful attention to the patient’s clinical presentation.

## Discussion

Rapid detection of SARS-CoV-2 is of utmost importance for maintaining a safe hospital environment with low rates of healthcare-associated infections. Indeed, rapid SARS-CoV-2 detection allows for implementing effective patient cohorting protocols and isolation of infected healthcare providers.

A similar study compared the sensitivity and specificity of rapid antigen test (RAT) versus qRT-PCR in 692 patients, the results showed that sensitivity of the antigen test was 63.5% compared to 100% specificity. The rapid antigen test showed poor outcomes in asymptomatic patients [2]. However, in another study RAT results vs. PCR were compared, and the sensitivity of RAT was higher than in the latter-mentioned study, sensitivity and specificity were 75.2% and 98.9%, respectively [3]. In this study, there were false negative results for symptomatic patients, which shows that RAT can’t be 100% reliable in the regards to patient isolation.

Another study reported the sensitivity of rapid antigen testing devices, the study included 412 patients, 11 patients had positive PCR and a negative rapid antigen test result. The sensitivity and specificity of the rapid antigen device was 79.06% and 100%, respectively [4]. Nagura-Ikeda and his colleagues, compared the sensitivity and specificity of 4 different tests in 102 patients’ samples of self-collected saliva, consisting of quantitative Reverse Transcription-PCR, Direct RT-PCR, Reverse Transcription–Loop-Mediated Isothermal Amplification, and Rapid Antigen Test and their final recommendation was against using rapid antigen test due to its low sensitivity [5]. In addition to another study using different testing methods, volume of samples, and dilution techniques has found that RAT could only identify 11.1% to 45.7% of the COVID-19-positive cases by RT-PCR [6].

As seen in Table 1, 34 of the cases detected as positive in RT-PCR were negative by RAT, on the other hand, 444 negative cases by PCR were also negative by RAT. Table 1 showed sensitivity and specificity of RAT in our study were 69.6% and 100%, respectively. Compared to other studies, the sensitivity value is similar and indicates that some positive cases could not be detected by the RAT.

Table 2 shows that patients with the highest viral load with a Ct value less than 20 had the highest detection values by the rapid antigen test, with a value of 91.8%. The World Health Organization (WHO) clearly stated that the Ct is inversely proportional to the viral load, which is also supported by our findings and other studies in the literature. And it’s also advised that another sample should be taken if the test results do not match the symptoms along with a new PCR [7]. Ciotti et al, tested 50 NPS, and concluded that the sensitivity of rapid antigen test was 30.77% and had 100% specificity, never the less, it was a significantly lower result than the sensitivity claimed by the manufacturer, this was explained by low Ct values, thus it is advised by WHO to use RAT with a sensitivity ≥80% and specificity ≥97% [8]. Another study, using the same Panbio rapid antigen test used in our research, reported that most of patients had a Ct value over 25, meaning that RAT missed 40% of the positive cases [9].

**Table 2:** Ct values and Panbio™ COVID-19 Ag rapid test results.

Ct	Antigen positive	Antigen negative	Total	Percent (%)
**Less than 20**	45	4	49	91.8
**20–29**	31	9	40	77.5
**30–34**	2	9	11	18.2
**35–40**	0	12	12	0

Table 3 indicates that the group with highest false-negative results was in the age group 41–50 years old. In another report by Tabain et al, false negative results were compared between different age groups, the highest false negative result was in the age group older than 65 years old [10]. Mostly, this can be attributed to Ct values distribution in different age groups in each study, and false negative results are not directly related to age groups.

**Table 3:** Relation between age groups and Ct values in false negative results in rapid antigen test.

**Age Group**	**Ct**					
		**Less than 20**	**20–29**	**30–34**	**35–40**	**Total**
	**0–10**					
	**11–20**				1	
	**21–30**	1	1	2		
	**31–40**	1	1	2	2	
	**41–50**	1	2	2	2	
	**51–60**		2		4	
	**61–70**	1	2	3	2	
	**71–80**		1		2	
	**81–90**					
	**Total**	4	9	9	12	34

Tests’ sensitivity can be decided based on their binding targets in some cases. RT-PCR tests target nonstructural proteins including RdRP, E, N, or S genes. Among these tests, the ones targeting RdRP gene were found to be with the highest analytical sensitivity [11].

There is a limitation for reverse transcription loop-mediated isothermal amplification RT-LAMP technique of false positive results when amplification time exceeds 30 minutes. Which also reduced the possibility of detecting low viral loads through this method. So, a primer was developed to reduce mi-amplification possibility til 120 minutes, which makes it a promising technique to be used by health workers and patients in hospital and home setting [12]. Also, this test can detect mutated strains of the virus, as well as a 94.6% sensitivity and 92.9% specificity. It does not need experienced operators nor special equipment [13]. In addition to this, RT-LAMP has been tested using fluorometric dye, and the accuracy of results was 94.03%, with an average reaction time of 26.2 minutes making it suitable for use outside hospitals and medical centers [14]. RT-qPCR is considered one of the mostly used tests, but its main disadvantage lies in the false negative results in low viral load samples [15].

In previous studies, it has been shown that COVID-19 may influence Angiotensin converting enzyme-2 (ACE-2) which can explain some of its cardiac, urinary, neurologic, respiratory, and reproductive system leading to multi-organ failure and septic shock in certain cases. One of the proposed mechanisms in urinary system involvement is damaging the kidneys through the virus binding to ACE-2 in the kidneys, as well as cytokine storms and hypoxemia that can all contribute to renal damage [16].

P-Value of 0.007 indicates that our hypothesis that the difference between rapid test results of COVID-19 samples versus PCR test is statistically significant. However, PCR testing is more sensitive and might be considered in rapid test negative cases if it is still suspected to have a positive result through PCR based on symptoms or exposure to infected individuals.

In our study, one of the main limitations was that symptoms were not reported for all cases, a previous study showed that RAT sensitivity was 34% in an emergency department with 421 patients participating in the study. On the other hand, sensitivity was 83% in symptomatic cases [17]. Most studies agree on the fact that RAT can be mostly reliable in patients with respiratory symptoms and not asymptomatic individuals [18].

## Conclusion

Based on the results obtained in this research, and other similar studies, it can be concluded that using qRT-PCR testing in asymptomatic patients is preferred, while in symptomatic patients antigen testing somehow showed positive results that can be used to make fast and effective decisions regarding the isolation of patients and preventing outbreaks in hospital setting, thus we recommend performing RT-PCR in patients with negative results that are highly suspected to have Covid-19 afterwards.

## Supporting information

S1 Data (XLSX)
